# Effect of Acupoint Catgut Embedding for Middle-Aged Obesity: A Multicentre, Randomised, Sham-Controlled Trial

**DOI:** 10.1155/2022/4780019

**Published:** 2022-02-27

**Authors:** Xia Chen, Wei Huang, Dan Wei, Ji-Ping Zhao, Wei Zhang, De-Guang Ding, Yang Jiao, Hong-Ling Pan, Jia-Jia Zhang, Feng Zhong, Feng Gao, Yi-Ting Jin, Yi-Wei Zheng, Yan-Ji Zhang, Qiao Huang, Xian-Tao Zeng, Zhong-Yu Zhou

**Affiliations:** ^1^Hubei University of Chinese Medicine, Wuhan, China; ^2^Affiliated Hospital of Hubei University of Chinese Medicine, Wuhan, China; ^3^Hubei Provincial Hospital of Traditional Chinese Medicine, Wuhan, China; ^4^Dongzhimen Hospital, Beijing University of Chinese Medicine, Beijing, China; ^5^The First Hospital of Hunan University of Chinese Medicine, Changsha, China; ^6^Center for Evidence-Based and Translational Medicine, Zhongnan Hospital of Wuhan University, Wuhan, China; ^7^Center for Evidence-Based and Translational Medicine, Wuhan University, Wuhan, China; ^8^Department of Evidence-Based Medicine and Clinical Epidemiology, The Second Clinical College, Wuhan University, Wuhan, China

## Abstract

**Objectives:**

This study aimed to examine the efficacy and safety of acupoint catgut embedding (ACE) for obesity over a 16-week treatment period using sham stimulation as the control.

**Methods:**

A multicenter, randomised, parallel, sham-controlled trial was conducted from February 10, 2017, to May 15, 2018. Men with waistlines ≥85 cm and women with ≥80 cm at three sites were randomised to receive eight sessions (over 16 weeks) of ACE (*n* = 108) or sham ACE (*n* = 108) with skin penetration at sham acupoints. The catgut was embedded once every two weeks using two alternating sets of acupoints. The follow-up lasted for an additional 24 weeks. The primary outcome was the percentage waistline reduction from baseline to week 16.

**Results:**

We included 216 individuals in the intention-to-treat analysis. At 16 weeks, the rate of waistline reduction was 8.80% (95% confidence interval (CI), 7.93% to 9.66%) in the ACE group and 4.09% (95% CI, 3.18% to 5.00%) in the sham control group, with a between-group difference of 4.71% (95% CI, 3.47% to 5.95%; *P* < 0.0001). This difference persisted throughout the entire follow-up period (between-group difference after 24-week additional weeks, 4.94% (95% CI, 3.58% to 6.30%); *P* < 0.001). The subgroup analyses of waistline by sex (male/female) revealed treatment effects of 1.93 (95% CI, −0.37 to 4.23, *P* = 0.1) in the male group and 3.19 (95% CI, 1.99 to 4.39, *P* < 0.001) in the female group. The adverse event analysis suggested that ACE and laboratory tests confirmed the safety of ACE. *Discussion*. ACE for 16 weeks could decrease the waistline and weight and was safe for the treatment of obesity. Further research is needed to evaluate the long-term efficacy and sex differences. This trial is registered with NCT02936973.

## 1. Introduction

Obesity is a chronic metabolic disease characterised by excessive accumulation or abnormal distribution of fat in the body. It is a risk factor for many diseases, including type 2 diabetes mellitus, hypertension, and certain cancers [1–3]. Research shows that the incidence of cardiovascular events in patients with excessive waistline is 2.1 times higher than that in patients with normal waistline [4]. Every 11 cm increase in waistline increases the risk of obesity-related cancer by 13% [5]. The number of obese people worldwide increased from 105 million in 1975 to 641 million in 2014 [6]. It has been projected that there will be 2.16 billion overweight and 1.12 billion obese individuals globally by 2030 [7].

Various strategies, including lifestyle interventions, drug therapy, and metabolic surgery, have been shown to be successful in managing global obesity epidemics [8]. However, many studies on lifestyle interventions alone have shown that maintaining weight loss is difficult [8–10]. Pharmacotherapy is associated with adverse reactions, and bariatric surgery is associated with a variety of complications [10, 11]. Complementary/alternative therapies, such as acupuncture, have been extensively used to treat obesity [12–14].

ACE is a combination of manual acupuncture and modern technologies that have evolved rapidly over the past 60 years. ACE involves infixing self-absorptive chromic surgical catgut sutures into acupoints and thus could provide the sensation of needling stimulation over a prolonged period of time (two to four weeks) [15]. The advantages of ACE are easy operation, time-saving, and durable stimulation, which increases patient compliance, compared with manual acupuncture [16, 17]. A meta-analysis of ACE for obesity [18] in 2014 suggested that ACE was more effective than manual acupuncture and equivalent to electro-acupuncture. Another meta-analysis in 2015 suggested that ACE is equivalent to pharmacotherapy in terms of the improvement of weight, BMI, waistline, and hipline [19].

A network meta-analysis of acupuncture and related therapies for obesity was conducted in 2018, and ACE was found to be better than sham acupuncture in reducing weight and BMI [20]. However, studies included in these meta-analyses are insufficiently powered and have high risks of bias and insufficient sample size. Randomised controlled trials with high quality and large sample sizes are required. Hence, a randomised, multicenter trial was conducted to examine the efficacy and safety of ACE for obesity over a 16-week treatment period using sham stimulation as the control.

## 2. Materials and Methods

### 2.1. Study Design

This was a multicentre, randomised, participant-blinded, sham-controlled trial. The participating sites included the Hubei Provincial Hospital of TCM (IRB Approval ID: HBZY2016-C20-01), Dongzhimen Hospital Beijing University of Chinese Medicine (IRB Approval ID: DZMEC-KY-2017-15), and the First Hospital of Hunan University of Chinese Medicine (IRB Approval ID: HN-LL-KY-2017-001-01). The study protocol was approved by the institutional review boards of the three hospitals [21]. Written informed consent was obtained from all participants. This trial was registered at ClinicalTrials.gov (NCT02936973).

### 2.2. Participants

Participants were eligible if they fulfilled the following criteria: age between 18 and 45 years, waistline ≥85 cm in men and waistline ≥80 cm in women [4], and a smoker who had not changed their smoking habits for at least two months [22]. Exclusion criteria included the following: endocrine disease (such as polycystic ovary syndrome, Cushing's disease, or hypothyroidism); type 2 diabetes mellitus or poorly managed hypertension (SBP ≥160 mmHg; DBP ≥100 mmHg) [23]; serious lung, heart, liver, or kidney disease; nervous system disease or mental disorders, history of depression that led to hospitalisation, two instances of suicidal attempt; clinical diagnosis of an eating disorder [24] such as bulimia [25], the term polyphagia or anorexia; weight changes greater than 5 kg in the previous three months; use of drugs with a known influence on weight or appetite in the previous three months, such as diet pills, corticosteroids, antidepressants, diazepam, nonselective antihistamines, nicotine replacements, or hypoglycaemic drugs or planning to give up smoking and drinking; pregnancy, lactation, or planning to become pregnant within 40 weeks; prior use of ACE; participation in clinical research on obesity in the previous three months; protein allergies and scar constitution; skin diseases such as eczema at the site of planned ACE and psoriasis; and coagulation disorders, or use of warfarin, heparin, or other anticoagulant drugs.

### 2.3. Randomisation and Masking

Participants were allocated to the ACE or sham ACE groups using stratified block randomisation. The randomisation sequence was generated using PROC PLAN in SAS (Version 9.4; SAS Institute Inc., Cary, NC) with the study site as the stratification factor and block size at 6. Acupuncturists obtained each patient's random number and assignment through an information management system for clinical scientific research. Each eligible patient received a group number according to the inclusion sequence and was assigned to the treatment program with the corresponding number, which remained the same throughout the trial.

Participants, evaluators, and statisticians, but not acupuncturists, were blinded to the treatment. In this trial, the acupuncturists placed the surgical catguts into embedding needles back to the participants; thus, the operation provided the participants blinding effects with a similar appearance to ACE, but no surgical catgut was embedded. A separate treatment room was used to make appointments with patients to minimise communication among the participants. To test participant blinding, all participants from three hospitals were asked to guess whether they had received ACE or sham ACE within 5 min after the treatment sessions on weeks 8 and 16.

### 2.4. Interventions

Standard operating procedures (SOPs) for all interventions and evaluations were formulated. ACE was performed by licensed acupuncturists with at least three years of clinical experience and lasted for 16 weeks. All researchers in the three hospitals received a one-day training session in Wuhan before recruitment.

**Figure 1:**
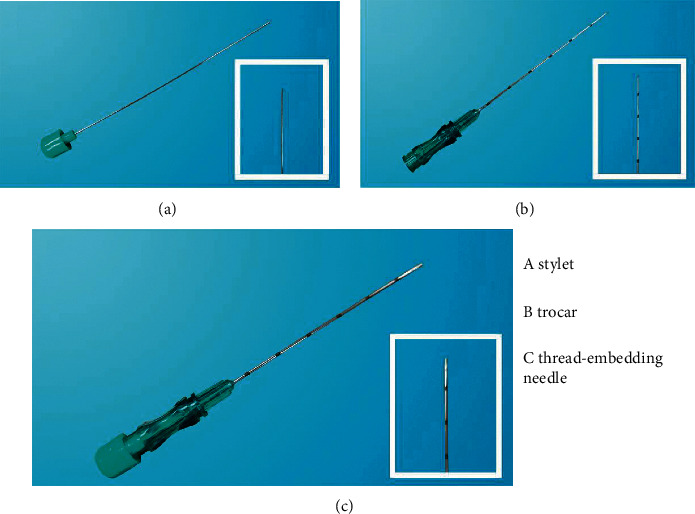
Stylet, trocar, and thread-embedding needle.

The acupoints were selected according to traditional Chinese medicine theory based on a review of the literature [26] and clinical expert consensus. The first set of acupoints included bilateral Zhiguo (TE6), bilateral Tianshu (ST25), bilateral Weishu (BL21), bilateral Zusanli (ST36), and Zhongwan (CV12). The second set of acupoints included bilateral Quchi (LI11), bilateral Huaroumen (ST24), bilateral Pishu (BL20), bilateral Fenglong (ST40), and Shuifen (CV9). Two groups of acupoints were used alternately once every two weeks. After disinfecting the acupoints, an absorbable surgical catgut suture (000, Shandong Boda Medical Supplies Co. Ltd., Shandong, China) 2 cm in length was placed at the front end of the trocar, the diameter of which was 0.8 mm, before the stylet was connected (Figure 1). A thread-embedding needle (#9#, Zhenjiang Gaoguan Medical Appliance Factory, Jiangsu, China) was inserted and advanced gradually until *deqi* (sensation of soreness, numbness, distention, or radiating) was reported by the participants upon lifting, trusting, twirling, and rotating the needle. The surgical catgut suture was pushed into the tissue using a stylet. Participants in the two groups received eight sessions of treatment (once every two weeks for 16 weeks), and each session lasted over 15 minutes.

Sham ACE was delivered to 1 cun (≈20 mm) [27] from the acupoints used in the ACE group, a location that did not represent any acupoints or meridians. The protocol in the sham ACE group was identical to that in the ACE group, with the exception that *deqi* sensation was not achieved and the surgical catgut was not embedded.

A diet and exercise diary and accompanying instruction manual were maintained, as described previously [28]. All participants were given diet and exercise education, and detailed guidance concerning their diet and exercise was provided using an instruction manual for dining and sports. Participants were asked to record their diet and exercise for at least four days per week (three days on weekdays and one day on the weekend). The caloric intake and consumption were estimated using a diary, which was collected at baseline and at weeks 4, 8, 12, 16, 28, and 40. Participants who took more calories than they consumed in daily life were classified as those who had a bad lifestyle, and further guiding suggestions were provided. Social tools (QQ and WeChat) for lifestyle modification were used to build a social network among the investigators and participants during the 16 weeks of treatment and 24 weeks of follow-up in the two groups.

### 2.5. Measurements

The primary outcome was the percent reduction in the waistline at 16 weeks relative to the pretreatment baseline. Secondary outcomes included percent reduction in weight, body mass index (BMI), hipline, waist-to-hip ratio (WHR), and percent body fat (PBF), as well as the change in the Impact of Weight on Quality of Life-Lite (IWQOL-Lite) scores, 36-Item Short Form (SF-36), Hospital Anxiety and Depression Scale (HAD), and Self-Esteem Scale (SES) scores at 16 weeks.

A complete physical examination including routine blood and hepatorenal function was conducted at baseline and at 16 weeks. Adverse events (AEs), including allergic reactions, syncope, local induration, local haematoma, and local infection, were assessed and recorded during the study. Pain associated with treatment was assessed using the visual analogue scale (VAS).

### 2.6. Statistical Analysis

Based on a previous study [29] and clinical practice, a 7% waistline reduction was anticipated at 16 weeks in the ACE group and a 2% reduction in the sham ACE group. Eighty-six participants per group were required based on (1) a mean clinically relevant difference of 5% (2% reduction in the sham ACE group), with a pooled standard deviation (SD) of 8.1%; and (2) two-sided at 5% and 1% at 80%. Assuming a 20% attrition rate, a total of 216 participants (108 per group) were planned for enrolment in this study.

Categorical variables were described using frequencies and percentages, continuous variables as means, or as median interquartile ranges if the data were skewed. Primary and secondary outcomes were evaluated in the intention-to-treat population. Missing data were imputed by multiple imputations (*m* = 5). The percentage of reduction from baseline ((before value − after value)/before value ∗ 100%) and change from baseline (after value - before value) as the dependent variables were analysed using a repeated-measures mixed-effects model with adjustment for baseline level, sex, age, marital status, family history of obesity, and duration of disease as fixed effects, while centre effect was assessed by including indicator of study sites as random effect for the G matrix. The differences between the two groups at each visit point were estimated by including treatment group, visit (categorical variable), interaction between treatment groups, and visit as fixed effect. An overall evaluation was performed to test the linear growth tendency by considering the visit as a continuous variable. The adjusted mean difference and 95% confidence intervals are presented.

Safety assessments were conducted using a safety analysis set. The incidence of AEs was compared using Fisher's exact method. The changes in laboratory parameters between baseline and 16 weeks were analysed using analysis of covariance. The McNemar test was employed to test inconsistencies in the grouping information, and blinding assessment was performed on each participant. A sensitivity analysis was conducted to assess the effect of missing data on the primary outcome. The data were transformed into a long format for each participant. In the long format, each row was one time point per participant. Therefore, each participant will have multiple rows of data. The rows with missing outcomes were excluded from the mixed-effects model.

All statistical analyses were conducted using a two-sided type I error rate of 5%. All analyses were carried out using SAS software, version 9.4 TS1M6 (SAS Institute Inc., Cary, NC). Data visualisation was conducted using R version 3.5.1 software (The R Foundation for Statistical Computing, Vienna, Austria; http://www.r-project.org/) with the R package ggplot2.

## 3. Results

### 3.1. Participants and Baseline Characteristics

**Figure 2:**
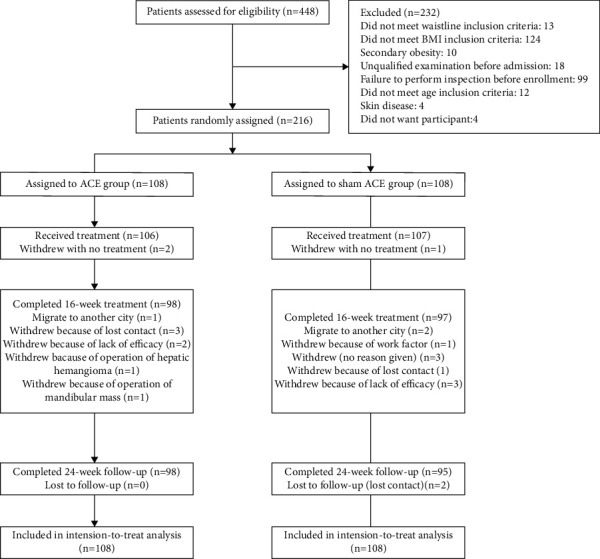
Flowchart of patient screening, enrolment, randomisation, and follow-up.

A total of 448 individuals were screened at three participating sites from February 10, 2017, to May 15, 2018. After 232 participants were excluded (Figure 2), a total of 216 individuals (108 in each group) were randomised. A total of 23 (10.6%) participants were lost to follow-up: 10 (9.3%) in the ACE group and 13 (12.0%) in the sham ACE group. The baseline demographic and clinical characteristics were similar between the two groups, except for a family history of obesity (Table 1).

### 3.2. Primary Outcome

**Figure 3:**
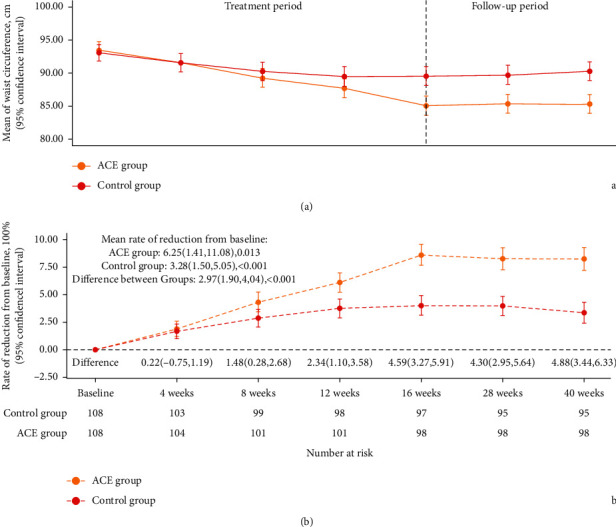
Change in waistline during the study.

At baseline, the median of waistline in the ACE group was 93.25 cm (IQR, 87.50, 99.00) compared with 92.00 cm (IQR, 88.00, 98.00) in the sham ACE group. The waistline reduction rate at 16 weeks was 8.80% (95% CI, 7.93%–9.66%) in the ACE group and 4.09% (95% CI, 3.18% to 5.00%) in the sham ACE group (between-group difference, 4.71% (95% CI, 3.47% to 5.95%); *P* < 0.001). The rate of waistline reduction at 40 weeks was 8.46% (95% CI, 7.52 to 9.40) in the ACE group and 3.52% (95% CI, 2.55 to 4.49) in the sham control group (between-group difference, 4.94% (95% CI, 3.58% to 6.30%); *P* < 0.001). The overall waistline reduction rate was 6.37% (95% CI, 1.42% to 11.33%) in the ACE group and 3.37% (95% CI, 1.62% to 5.12%) in the sham ACE group (between-group difference, 3.00% (95% CI, 1.99% to 4.02%); *P* < 0.001).  Table 2 summarises the waistline rate of reduction from baseline over 40 weeks. The sensitivity for the primary outcome was similar between the groups (Table 3).  Figure 3 shows the changes in waistline in the groups during the study. Compared with the sham ACE group, participants in the ACE group had greater waistline reduction at all time points.

This study used a repeated-measures design. A number of participants dropped out gradually during the follow-up period (4 weeks, 4 and 5; 8 weeks, 3 and 4; 12 weeks, 1 and 1; 16 weeks, 2 and 1; 28 weeks, 0 and 2; in the ACE and sham ACE groups, respectively). At the end of follow-up (40 weeks), 10 and 13 participants were missing in the ACE group and in the sham ACE group, respectively. Meanwhile, the number of complete data points at each time point is presented at the bottom of Figure 3. Processing of the missing data in the primary outcome followed a prespecified statistical analysis plan. About 10% of missing data were presented, and multiple imputation was applied using fully conditional specification, implemented with SAS 9.4 procedure multiple imputation (PROC MI) under the fully conditional specification (FCS) option. The 23 participants' last observed measures and baseline characteristics were used for prediction in imputation.

The sensitivity analyses were performed to assess the impact of missing data on the primary outcome. The results of multiple imputation and complete data are summarised in Tables 2 and 3. The overall adjusted difference was 3.00 (95% CI, 1.99 to 4.02) in the intention-to-treat (ITT) analysis and 3.18 (95% CI, 2.18 to 4.18) in the complete data. In ITT analysis, the estimate of the treatment effect tended to be conservative because of dilution due to noncompliance. The findings using complete cases did not change substantially and seemed to be robust about missing data.

The two-level interaction between sex and treatment group, two-level interaction between sex and visit time point, and three-level interaction among sex, treatment group, and visit time point were introduced in the repeated-measures mixed-effects model, and their *P* values were estimated using type 3 fixed-effects tests. Meanwhile, the subgroup analyses were performed according to sex (male/female). In the primary analysis on percentage change in waistline, the treatment effect (differences in change percentage between the two treatment groups) was 1.93 (95% CI, -0.37 to 4.23, *P* = 0.1) in the male group and 3.19 (95% CI, 1.99 to 4.39, *P* < 0.001) in the female group based on the subgroup analysis. Wide overlap between the two CIs was observed, and *P* values for the interaction between sex and group and the interaction among sex, group, and visit time were 0.66 and 0.74, respectively. This suggests the homogeneity of the treatment effect. A significant effect was found in the female group and not in the male group. The results are shown in Table 4. The changes in absolute values for waistline were similar between groups (Tables 5 and 6).

### 3.3. Secondary Outcomes

Table 7 summarises the secondary outcomes. ACE was associated with greater reduction rates from baseline than placebo in weight (7.92% with ACE vs. 2.91% with placebo; between-group difference, 5.01%; 95% CI, 3.74% to 6.29%; *P* < 0.001), BMI (7.93% with ACE vs. 3.06% with placebo; between-group difference, 4.87%; 95% CI, 3.71% to 6.03%; *P* < 0.001), hipline (3.73% with ACE vs. 1.88% with placebo; between-group difference, 1.85%; 95% CI, 1.02% to 2.68%; *P* < 0.001), WHR (4.97% with ACE vs. 1.77% with placebo; between-group difference, 3.20%; 95% CI, 2.06% to 4.35%; *P* < 0.001), and PBF (10.01% with ACE vs. 5.01% with placebo; between-group difference, 5.00%; 95% CI, 3.07% to 6.93%; *P* < 0.001). The group differences in weight, BMI, hipline, WHR, and PBF remained statistically significant during the 24 weeks of follow-up (*P* < 0.001). Both groups showed improvements in SF-36, HAD, and SES over 1 to 16 weeks, without significant differences between the two groups (*P* > 0.05). Benefits favouring ACE were also noted with respect to changes in IWQOL-Lite at week 40 compared with the sham ACE group (*P* < 0.001). The results of the subgroup analyses were summarised by sex (male/female) for all secondary outcomes, and all *P* values for the interaction between sex and group and the interaction among sex, group, and visit time were greater than 0.05. The results of the change in absolute values for weight, BMI, hipline, WHR, PBF, SF-36, HAD, and SES IWQOL-Lite were similar between groups (Table 8).  Table 9 shows the baseline values and the values of the end of study (week 16 and week 40).

### 3.4. Blinding

At week 8, 93 of 101 participants in the ACE group vs. 89/99 participants in the sham ACE group guessed their treatment to be ACE. At week 16, the numbers were 92/98 participants in the ACE group vs 89/97 participants in the sham ACE group. The statistical significance at both week 8 and week 16 and similar percentages in guessing that they received ACE in two groups supported blindness were successful in the trial (*P* < 0.001) (Table 10).

### 3.5. Safety

The most common ACE-related AEs were haematoma around the site of needling in the ACE group (1.9% vs. 0 in the sham ACE group). Adverse events related to treatment occurred in 7.5% and 4.7% of participants in the ACE and sham ACE groups, respectively. Neither group had severe AEs (Table 11). No AEs necessitated the withdrawal of participants from the trial. At 16 weeks, routine blood and hepatorenal function measures were not significantly different between the two groups (Table 12).

## 4. Discussion

To our knowledge, this study is the largest multicentre, sham-controlled clinical trial to assess the beneficial effect of ACE on obesity within 16 weeks. Repeated ACE was found to reduce waistline in obese patients by an average of 8.80% after 16 weeks of treatment. This effect persisted for at least 24 weeks after discontinuation of the treatment.

ACE refers to the procedure of embedding sutures made of absorbable materials into the skin tissue of acupoints, which are closely related to different physiological processes or diseases. It exhibited a therapeutic effect on chronic diseases by dredging the channels, invigorating the pulse, and regulating Qi and blood. ACE is a subtype of acupuncture that can extend the sensation of needling. Recent evidence suggests that the experimental mechanism of acupuncture in obesity is mainly focused on the central nervous system and peripheral adipose tissue [30]. The probable mechanism by which both ACE and electro-acupuncture have positive effects in reducing weight in obese patients may be related to its effects in downregulating serum leptin and insulin levels and correcting leptin resistance and insulin resistance [31]. Due to the small sample size and lack of sham control, further research is needed.

ACE was chosen for obesity instead of acupuncture due to the following reasons. First, ACE requires a lower frequency of treatment than acupuncture. For chronic diseases such as obesity, acupuncture must be conducted every day or every other day in China, while ACE treatment can be administered once every two to four weeks [31, 32]. Second, the merit of ACE therapy is the more continuous stimulation of the catgut than acupuncture needling on acupoints. Meta-analysis showed that ACE is more effective than acupuncture in the treatment of obesity [17, 32]. Third, the cost-effective analysis showed that the cost per patient in the ACE treatment was less than that in the acupuncture treatment [33]. The above results indicate that ACE has a significant effect on obesity with low cost and fine economic benefit.

In this study, ten acupoints were selected for treatment. Based on Chinese traditional medicinal theory, the acupuncture treatment of obesity focuses on regulating the spleen and stomach, which is closely correlated with the Taiyin spleen channel of the foot, the Yangming stomach channel of foot, and the Ren channel. Thus, the combination of specific acupoints for these three meridians was selected in this trial according to a previous review of ancient and modern literature.

The study included a sham control group. The surgical catgut was not embedded in the sham group to avoid long-term stimulation at the acupoint. Sham acupoints were delivered at the point 1 cun near the actual acupoint, and the location did not represent any acupoints or meridians, which minimised any physiological effect in the sham ACE group. Considering the possible blind destruction of the occurrence of AEs related to catgut embedding treatment, people who had used ACE therapy before were excluded from this study. To improve blinding and participant adherence, all individuals visited the hospital separately to avoid communication among the participants. The blinding assessment results suggested that the blinding was successful. In the study, the two groups received the same diet and exercise education, so the sham ACE group also had a reduction in the waistline.

In this study, patients aged >45 years were excluded, since acupuncture or ACE is less effective in elderly patients with multiple comorbidities. Patients aged between 18 and 45 were enrolled to ensure similar baseline characteristics of the patients and to observe the efficacy of ACE for obesity. From the authors' experience, ACE alone is not suitable for elderly patients with obesity. ACE combined with moxibustion is a common supplementary therapy to improve the weight loss effect in elderly obesity. More research should be conducted to confirm a suitable therapy for elderly patients with obesity.

In a previous ACE study in obese women [34], surgical catgut was stimulated at CV6, CV9, ST28, KI14, and ST36, and the differences between active ACE and the placebo in mean waistline reduction from the baseline were 4.84 cm vs. 1.68 cm (*P* < 0.001) after six weeks of treatment of one embedding per week. The difference in mean weight reduction was 1.65 kg vs. 0.38 kg (*P* < 0.001). However, the study had limitations of sample size and single centre and did not examine whether the ACE effect lasted after discontinuation after the treatment. In this study, obese patients were enrolled as the research target, and different acupoints were chosen. It was observed that the waistline reduction rate was 8.46% (95% CI, 7.52% to 9.40%) in the ACE group and 3.52% (95% CI, 2.55% to 4.94%) in the sham ACE group after 24 weeks without ACE treatment. These results showed that ACE may have a long-term therapeutic effect in obese patients.

Recent studies have demonstrated that waistline and WHR are better obesity indicators for Chinese people than BMI [35–37]. Thus, waistline was chosen as the diagnosis and primary outcome instead of weight or BMI. In this study, individuals whose waistline was ≥85 in men and waistline ≥80 in women were included. Based on *the Guidelines for Prevention and Control of Overweight and Obesity in Chinese Adults* published by the Department of Disease Control Ministry of Health of China, men with a waistline >85 cm and women with a waistline of >80 cm are 3.5 times more likely to suffer from hypertension than those below these values and that the risk of diabetes is approximately 2.5 times [4]. In this trial, adults with obesity (waistline ≥85 in men and waistline ≥80 in women) and without metabolic diseases had a mean waistline loss of 8.80% from baseline with ACE as an adjunct to lifestyle intervention after 16 weeks. This loss exceeded that of placebo plus lifestyle intervention by 4.71% points. This suggests that ACE may be beneficial for metabolic syndrome because it improves abdominal adiposity. The second outcome of BMI, weight, hipline, WHR, and PBF in this study further revealed that ACE could be an effective treatment for obese patients. This study identified a trend for the improvement of IWQOL-Lite, SF-36, HAD, and SES following ACE treatment. The statistical superiority of ACE over placebo was not achieved for all scales. This is likely due to the study's enrolment participants being in the mild category of obese who had slightly related indicators. Thus, there remains a need for additional large-scale clinical trials to further investigate the effects of ACE on these indicators. The continuous outcome can be analysed using the actual value, change from baseline, and percentage change from baseline. Medical intervention is expected to present changes in a condition. In this study, an analysis of both changes from baseline and percentage change from baseline was performed to improve the understanding of the results. The analysis results of the change from baseline and percentage change from baseline were similar.

In the subgroup analysis, a significant effect was found in the female group and not in the male group. This does not mean that the effect was different between the two subgroups. Statistical significance was greatly associated with the sample size. In this study, the sample size estimation was performed without considering sex differences. Fifty-one males (28 in the ACE group and 23 in the sham ACE group) and 165 females (80 in the ACE group and 85 in the sham ACE group) were recruited. The relatively small sample size in the male group would lead to insufficient power to detect the treatment effect in this group. *P* values of both primary outcome and secondary outcomes for the interaction between sex and group and the interaction among sex, group, and visit time were greater than 0.05. Although no significant modification effect of sex was found in this study, a certain degree of difference between the two-point estimations of treatment effect from the male and female groups provided a meaningful clue for further study.

In this study, the proportion of participants with ACE-related AEs in the ACE group was low, which was also observed in other ACE-related studies [38, 39]. It took about two weeks for the catgut and local induration to be completely absorbed. To better self-absorb catgut, the alternation of acupoint stimulation was chosen, which has been used in a previous study, and contributed to weight loss [31]. Haematoma around the site of needling, sleeplessness, dizziness, and fainting were exhibited by only a few individuals, which were similar to those reported in earlier conventional acupuncture and ACE-related studies [27, 32]. No serious AEs were observed during the trial. The results of routine laboratory measurements before and after treatment provided further reassurance of the safety of ACE in the treatment of obesity. The dropout rate in other clinical studies on obesity was approximately 10% [40, 41], and the dropout rate was 10.6% in this study. This is attributed to the difficulty in managing the obese population at long treatment and follow-up periods. In fact, the trial lasted for 40 weeks. Most participants discontinued the study for personal reasons instead of AEs. This study provides additional reassurance regarding the safety of ACE in the treatment of obesity.

### 4.1. Strengths and Limitations

The strengths of this trial included the large sample size, high rates of adherence to the treatment regimen, and completion of the trial. This is the first study to provide a subgroup analysis of curative effect differences for ACE between men and women, and the results can provide meaningful clues for further study. This study has several limitations. First, long-term follow-up was not assessed. The session and follow-up in this study were based on expert consensus in China. However, a longer-term follow-up may be needed for a forward observation of whether obesity regains in the target crowd. Second, a control group of diet and exercise only or waiting list groups was not included in the research. This deserves further study in the future. Finally, the frequency, duration, length of surgical catgut, and selection of acupoints in ACE treatment warrant further investigation.

## 5. Conclusions

The 16-week ACE treatment decreased waistline, BMI, weight, hipline, WHR, and PBF in obese individuals and is safe for treatment. The effect persisted for at least 24 weeks after treatment discontinuation.

## Figures and Tables

**Table 1:** Participant baseline characteristics.

Characteristics	ACE group (*n* = 108)	Sham ACE group (*n* = 108)
*Sex*		
Male, *n* (%)	28 (25.93%)	23 (21.30%)
Female, *n* (%)	80 (74.07%)	85 (78.70%)
Age, mean ± SD, y	31.66 ± 6.55	30.75 ± 6.71
Married, *n* (%), yes	71 (65.74%)	63 (58.33%)
Father or mother obesity, *n* (%), yes	79 (73.15%)	91 (84.26%)
Duration, median (IQR) (cm)	79.50 (44.50, 126.00)	67.00 (36.00, 120.00)
Waistline, median (IQR) (cm)	93.25 (87.50, 99.00)	92.00 (88.00, 98.00)
Weight, median (IQR) (kg)	72.90 (67.45, 80.25)	72.00 (66.30, 80.60)
BMI, median (IQR) (kg/m^2^)	27.42 (25.97, 28.89)	27.49 (25.95, 28.93)
Hipline, median (IQR) (cm)	104.00 (100.00, 108.00)	103.00 (100.00, 107.50)
WHR, median (IQR)	90.00 (85.00, 95.00)	90.00 (86.00, 94.00)
PBF, median (IQR) (%)	33.10 (30.25, 35.10)	33.15 (30.50, 35.35)
IWQOL-Lite, median (IQR)	58.00 (44.50, 72.00)	53.50 (44.00, 66.50)

*SF-36, median (IQR)*		
PF	95.00 (85.00, 95.00)	95.00 (90.00, 95.00)
RP	100.00 (75.00, 100.00)	100.00 (75.00, 100.00)
BP	72.00 (62.00, 100.00)	72.00 (62.00, 100.00)
GH	62.00 (48.50, 76.00)	67.00 (57.00, 77.00)
VT	75.00 (65.00, 80.00)	75.00 (65.00, 80.00)
SF	100.00 (87.50, 112.50)	100.00 (87.50, 112.50)
RE	100.00 (66.67, 100.00)	100.00 (66.67, 100.00)
MH	72.00 (64.00, 80.00)	72.00 (64.00, 80.00)

*HAD, median (IQR)*		
Anxiety	4.00 (2.00, 6.00)	4.00 (3.00, 6.00)
Depression	4.00 (2.00, 6.00)	3.00 (2.00, 5.50)
SES, median (IQR)	33.00 (30.00, 35.00)	32.50 (29.00, 36.50)

ACE, acupoint catgut embedding; sham ACE, sham acupoint catgut embedding; BMI, body mass index; PBF, percent body fat; WHR, waist-to-hip ratio; IWQOL-Lite, the Impact of Weight on Quality of Life Questionnaire; SF-36, 36-Item Short Form; HAD, the Hospital Anxiety and Depression Scale; SES, the Self-Esteem Scale; IQR, interquartile range.

**Table 2:** Percentage reduction in waistline (%) based on intention-to-treat principle with multiple imputation.

Time point	Percentage of reduction from baseline (%) Adjusted mean (95% (confidence interval), *P*			Mixed-effects model using visit as continuous variable Coefficient (95% confidence interval), *P*		
	ACE group	Sham ACE group	Difference	Group	Time	Group^a^ Time
4 weeks	1.95 (1.31, 2.60), <0.001	1.78 (1.13, 2.44), <0.001	0.17 (-0.74, 1.08), 0.718			
8 weeks	4.34 (3.56, 5.13), <0.001	2.96 (2.19, 3.73), <0.001	1.38 (0.29, 2.48), 0.014			
12 weeks	6.20 (5.35, 7.05), <0.001	3.84 (2.99, 4.70), <0.001	2.36 (1.16, 3.56), <0.001			
16 weeks	8.80 (7.93, 9.66), <0.001	4.09 (3.18, 5.00), <0.001	4.71 (3.47, 5.95), <0.001	0.86 (-0.88, 2.60), 0.333	0.01 (-0.01, 0.03), 0.279	0.07 (0.04, 0.10), <0.001
28 weeks	8.49 (7.62, 9.36), <0.001	4.02 (3.12, 4.92), <0.001	4.46 (3.21, 5.72), <0.001			
40 weeks	8.46 (7.52, 9.40), <0.001	3.52 (2.55, 4.49), <0.001	4.94 (3.58, 6.30), <0.001			
Overall^b^	6.37 (1.42, 11.33), 0.013	3.37 (1.62, 5.12), <0.001	3.00 (1.99, 4.02), <0.001			

Percentage of reduction from baseline = (before value − after value)/before value ^∗^ 100%. ^a^Adjusted for baseline, sex, age, married status, duration of disease, and family history. ^b^The overall waistline reduction rate, namely the average percentage decrease during 0 to 40 weeks.

**Table 3:** Results of the sensitivity for the primary outcome.

Time point	Percentage of reduction from baseline (%) Adjusted mean (95% confidence interval), *P*			Continuous time in mixed-effects model Coefficient (95% confidence interval), *P*		
	ACE group	Sham ACE group	Difference	Group	Time	Group ^*∗*^Time
4 weeks	1.90 (1.26, 2.54), <0.001	1.80 (1.14, 2.46), <0.001	0.10 (-0.81, 1.02), 0.824			
8 weeks	4.37 (3.59, 5.15), <0.001	2.92 (2.12, 3.73), <0.001	1.45 (0.33, 2.57), 0.012			
12 weeks	6.20 (5.37, 7.03), <0.001	3.77 (2.92, 4.62), <0.001	2.43 (1.24, 3.62), <0.001			
16 weeks	8.93 (8.08, 9.78), <0.001	3.87 (3.01, 4.74), <0.001	5.06 (3.84, 6.28), <0.001	0.88 (-0.84, 2.61), 0.313	0.01 (-0.01, 0.03), 0.443	0.08 (0.05, 0.12), <0.001
28 weeks	8.58 (7.72, 9.45), <0.001	3.85 (2.97, 4.74), <0.001	4.73 (3.50, 5.96), <0.001			
40 weeks	8.58 (7.67, 9.49), <0.001	3.25 (2.32, 4.19), <0.001	5.33 (4.02, 6.63), <0.001			
Overall	6.43 (5.74, 7.12), <0.001	3.25 (2.53, 3.96), <0.001	3.18 (2.18, 4.18), <0.001			

Percentage of reduction from baseline = (before value − after value)/before value ^∗^ 100%.

**Table 4:** Subgroup analysis of percentage reduction in waistline (%) by the male group and the female group based on the intention-to-treat principle.

Time point	Percentage of reduction from baseline (%) in the male group (*n* = 51) Adjusted mean (95% confidence interval), *P*			Percentage of reduction from baseline (%) in the female group (*n* = 165) Adjusted mean (95% confidence interval), *P*		
	ACE group	Sham ACE group	Difference	ACE group	Sham ACE group	Difference
4 weeks	1.62 (0.47, 2.77), 0.006	1.55 (0.23, 2.87), 0.021	0.07 (-1.76, 1.91), 0.936	1.96 (1.13, 2.79), <0.001	1.81 (1.02, 2.59), <0.001	0.15 (-0.98, 1.28), 0.791
8 weeks	3.45 (2.04, 4.86), <0.001	2.79 (1.15, 4.43), <0.001	0.66 (-1.63, 2.96), 0.568	4.55 (3.54, 5.56), <0.001	2.98 (2.01, 3.96), <0.001	1.57 (0.20, 2.94), 0.025
12 weeks	5.22 (3.20, 7.23), <0.001	3.81 (1.50, 6.13), 0.001	1.40 (-1.80, 4.60), 0.389	6.35 (5.30, 7.39), <0.001	3.98 (3.06, 4.89), <0.001	2.37 (1.04, 3.70), <0.001
16 weeks	6.81 (5.05, 8.57), <0.001	3.59 (1.61, 5.57), <0.001	3.23 (0.57, 5.88), 0.017	9.23 (8.11, 10.35), <0.001	4.20 (3.19, 5.20), <0.001	5.03 (3.57, 6.49), <0.001
28 weeks	6.42 (4.52, 8.32), <0.001	3.57 (1.37, 5.76), 0.002	2.86 (-0.09, 5.80), 0.057	8.96 (7.83, 10.09), <0.001	4.25 (3.24, 5.26), <0.001	4.71 (3.21, 6.20), <0.001
40 weeks	6.41 (4.31, 8.51), <0.001	3.06 (0.72, 5.40), 0.011	3.35 (0.11, 6.60), 0.043	8.91 (7.76, 10.06), <0.001	3.58 (2.52, 4.64), <0.001	5.33 (3.80, 6.85), <0.001
Overall	4.99 (1.00, 8.98), 0.015	3.06 (0.65, 5.47), 0.013	1.93 (-0.37, 4.23), 0.100	6.66 (1.35, 11.97), 0.015	3.47 (1.57, 5.36), <0.001	3.19 (1.99, 4.39), <0.001

Percentage of reduction from baseline = (before value − after value)/before value ^∗^ 100%. ^a^*P* value for interaction between sex and group, and interaction among sex, group, and visit time was 0.66 and 0.74, respectively. ^b^Adjusted for baseline, age, married status, drinking and smoking status, duration of disease, and family history.

**Table 5:** Reduction in waistline from baseline (cm) based on intention-to-treat principle.

Time point	Reduction from baseline (cm) Adjusted mean (95% confidence interval), *P*			Mixed-effects model using visit as continuous variable Coefficient (95% confidence interval), *P*		
	ACE group	Sham ACE group	Difference	Group	Time	Group^a^ Time
4 weeks	−1.83 (−2.43, −1.24), <0.001	−1.64 (−2.25, −1.04), <0.001	−0.19 (−1.03, 0.65), 0.655			
8 weeks	−4.08 (−4.81, −3.35), <0.001	−2.75 (−3.47, −2.04), <0.001	−1.33 (−2.35, −0.30), 0.011			
12 weeks	−5.80 (−6.59, −5.00), <0.001	−3.57 (−4.36, −2.78), <0.001	−2.23 (−3.35, −1.11), <0.001			
16 weeks	−8.21 (−9.02, −7.40), <0.001	−3.81 (−4.66, −2.96), <0.001	−4.40 (−5.56, −3.24), <0.001	−0.83 (−2.43, 0.77), 0.308	−0.01 (−0.03, 0.01), 0.233	−0.07 (−0.10, −0.04), <0.001
28 weeks	−7.95 (−8.76, −7.13), <0.001	−3.76 (−4.60, −2.91), <0.001	−4.19 (−5.37, −3.01), <0.001			
40 weeks	−7.95 (−8.83, −7.06), <0.001	−3.29 (−4.20, −2.39), <0.001	−4.66 (−5.93, −3.38), <0.001			
Overall	−5.97 (−10.61, −1.33), 0.013	−3.14 (−4.78, −1.50), <0.001	−2.83 (−3.78, −1.89), <0.001			

Change from baseline: after value (posttreatment)–before value (baseline).

**Table 6:** Reduction in waistline from baseline in the male group and the female group based on the intention-to-treat principle.

Time point	Reduction from baseline (cm) in the male group (*n* = 51) Adjusted mean (95% confidence interval), *P*			Reduction from baseline (cm) in the female group (*n* = 165) Adjusted mean (95% confidence interval), *P*		
	ACE group	Sham ACE group	Difference	ACE group	Sham ACE group	Difference
4 weeks	−1.64 (−2.76, −0.53), 0.004	−1.58 (−2.86, −0.29), 0.016	−0.06 (−1.84, 1.71), 0.943	−1.81 (−2.56, −1.06), <0.001	−1.64 (−2.35, −0.93), <0.001	−0.17 (−1.19, 0.86), 0.749
8 weeks	−3.47 (−4.83, −2.12), <0.001	−2.86 (−4.47, −1.25), <0.001	−0.61 (−2.84, 1.62), 0.589	−4.20 (−5.12, −3.27), <0.001	−2.73 (−3.62, −1.84), <0.001	−1.46 (−2.72, −0.21), 0.022
12 weeks	−5.25 (−7.22, −3.27), <0.001	−3.87 (−6.16, −1.58), <0.001	−1.38 (−4.52, 1.76), 0.388	−5.81 (−6.75, −4.86), <0.001	−3.65 (−4.48, −2.82), <0.001	−2.16 (−3.36, −0.95), <0.001
16 weeks	−6.85 (−8.58, −5.13), <0.001	−3.65 (−5.60, −1.70), <0.001	−3.21 (−5.81, −0.60), 0.016	−8.44 (−9.47, −7.42), <0.001	−3.86 (−4.78, −2.95), <0.001	−4.58 (−5.92, −3.25), <0.001
28 weeks	−6.50 (−8.34, −4.66), <0.001	−3.62 (−5.77, −1.48), <0.001	−2.88 (−5.74, −0.01), 0.049	−8.22 (−9.27, −7.18), <0.001	−3.92 (−4.85, −2.99), <0.001	−4.30 (−5.69, −2.92), <0.001
40 weeks	−6.51 (−8.54, −4.49), <0.001	−3.14 (−5.41, −0.86), 0.007	−3.38 (−6.52, −0.24), 0.035	−8.20 (−9.27, −7.13), <0.001	−3.31 (−4.28, −2.34), <0.001	−4.89 (−6.31, −3.48), <0.001
Overall b	−5.04 (−9.05, −1.03), 0.015	−3.12 (−5.51, −0.73), 0.011	−1.92 (−4.14, 0.31), 0.091	−6.11 (−10.98, −1.25), 0.015	−3.19 (−4.94, −1.43), <0.001	−2.93 (−4.02, −1.83), <0.001

Change from baseline: after value (posttreatment)–before value (baseline). *P* value for interaction between sex and group, and interaction among sex, group, and visit time was 0.64 and 0.87, respectively. Adjusted for baseline, age, married status, drinking and smoking status, duration of disease, and family history.

**Table 7:** Secondary outcomes (percentage change from baseline and change from baseline) based on the intention-to-treat principle.

	Adjusted mean^1^			Difference (95% CI), *P*		*P* ^2^	*P* ^3^
	ACE group (95% CI)	Sham ACE group (95% CI)	Difference (95% CI), *P*	Male group (*n* = 51)	Female group (*n* = 165)		
*Weight (%)*						0.51	0.26
Week 16	7.92 (7.10, 8.74), <0.001	2.91 (2.00, 3.82), <0.001	5.01 (3.74, 6.29), <0.001	2.50 (−0.58, 5.57), 0.111	5.47 (4.07, 6.87), <0.001		
Week 40	7.28 (6.38, 8.19), <0.001	2.47 (1.40, 3.54), <0.001	4.81 (3.32, 6.31), <0.001	2.16 (−1.28, 5.61), 0.218	5.22 (3.65, 6.79), <0.001		
Overall effect	5.70 (1.42, 9.99), 0.011	2.32 (1.07, 3.57), <0.001	3.39 (2.41, 4.36), <0.001	1.63 (−0.78, 4.04), 0.185	3.69 (2.64, 4.75), <0.001		

*BMI (%)*						0.72	0.23
Week 16	7.93 (7.10, 8.75), <0.001	3.06 (2.25, 3.87), <0.001	4.87 (3.71, 6.03), <0.001	3.05 (0.67, 5.42), 0.012	5.54 (4.15, 6.93), <0.001		
Week 40	7.31 (6.38, 8.24), <0.001	2.73 (1.81, 3.65), <0.001	4.58 (3.26, 5.90), <0.001	2.75 (0.23, 5.28), 0.032	5.22 (3.77, 6.66), <0.001		
Overall effect	5.70 (1.39, 10.01), 0.011	2.48 (1.20, 3.77), <0.001	3.22 (2.32, 4.11), <0.001	2.05 (0.29, 3.81), 0.022	3.71 (2.68, 4.74), <0.001		

*Hipline (%)*						0.94	0.99
Week 16	3.73 (3.15, 4.30), <0.001	1.88 (1.30, 2.46), <0.001	1.85 (1.02, 2.68), <0.001	1.09 (−0.67, 2.85), 0.224	2.17 (1.19, 3.14), <0.001		
Week 40	3.71 (2.98, 4.45), <0.001	1.64 (0.89, 2.40), <0.001	2.07 (1.04, 3.10), <0.001	1.62 (−1.24, 4.49), 0.266	2.16 (1.02, 3.30), <0.001		
Overall effect	2.72 (0.54, 4.91), 0.016	1.46 (0.39, 2.53), 0.008	1.27 (0.62, 1.91), <0.001	0.71 (−0.69, 2.11), 0.323	1.50 (0.77, 2.24), <0.001		

*WHR (%)*						0.34	0.39
Week 16	4.97 (4.18, 5.76), <0.001	1.77 (0.92, 2.62), <0.001	3.20 (2.06, 4.35), <0.001	2.90 (0.72, 5.09), 0.009	2.79 (1.41, 4.17), <0.001		
Week 40	4.63 (3.74, 5.52), <0.001	1.43 (0.53, 2.33), 0.002	3.20 (1.93, 4.47), <0.001	2.28 (−0.51, 5.07), 0.109	2.93 (1.38, 4.48), <0.001		
Overall effect	3.45 (0.58, 6.31), 0.019	1.64 (0.62, 2.65), 0.002	1.81 (0.88, 2.74), <0.001	1.51 (−0.35, 3.37), 0.112	1.50 (0.40, 2.59), 0.007		

*PBF (%)*						0.79	0.64
Week 16	10.01 (8.66, 11.36), <0.001	5.01 (3.59, 6.43), <0.001	5.00 (3.07, 6.93), <0.001	3.70 (−2.65, 10.05), 0.247	5.61 (3.62, 7.61), <0.001		
Week 40	8.08 (6.56, 9.61), <0.001	3.71 (2.13, 5.29), <0.001	4.37 (2.14, 6.60), <0.001	3.08 (−3.35, 9.51), 0.344	5.08 (2.85, 7.31), <0.001		
Overall effect	6.92 (2.25, 11.59), 0.005	3.98 (1.77, 6.18), <0.001	2.94 (1.41, 4.48), <0.001	2.59 (−2.31, 7.50), 0.298	3.16 (1.69, 4.63), <0.001		

*IWQOL-Lite*						0.96	0.25
Week 16	−11.19 (−13.68, −8.71), <0.001	−8.96 (−11.66, −6.25), <0.001	−2.24 (−6.05, 1.58), 0.248	2.43 (−3.96, 8.81), 0.456	−3.46 (−7.67, 0.74), 0.106		
Week 40	−16.72 (−19.15, −14.29,<0.001	−10.37 (−12.75, −7.98), <0.001	−6.35 (−9.72, −2.99), <0.001	0.20 (−5.02, 5.42), 0.940	−8.42 (−12.37, −4.47), <0.001		
Overall effect	−10.73 (−19.44, −2.03), 0.017	−8.13 (−13.22, −3.05), 0.002	−2.60 (−5.14, −0.06), 0.045	1.48 (−2.30, 5.26), 0.443	−3.81 (−6.80, −0.82), 0.013		
SF-36 score	—	—	—				

*SF-36 (PF)*						0.20	0.65
Week 16	2.90 (1.12, 4.68)	0.93 (−0.85, 2.71)	1.97 (−0.54, 4.48), 0.124	−1.85 (−5.53, 1.83), 0.323	3.04 (−0.00, 6.09), 0.050		
Week 40	4.45 (2.89, 6.00)	2.92 (1.36, 4.49)	1.53 (−0.70, 3.75), 0.180	−0.85 (−6.09, 4.38), 0.748	2.40 (−0.14, 4.94), 0.064		
Overall effect	3.67 (1.56, 5.79)	1.93 (−0.48, 4.34)	1.75 (−0.43, 3.92), 0.115	−1.35 (−5.27, 2.57), 0.497	2.72 (0.15, 5.29), 0.038		

*SF-36 (RP)*						0.59	0.52
Week 16	3.52 (−0.53, 7.56)	5.91 (2.29, 9.52)	−2.39 (−8.04, 3.26), 0.404	−2.34 (−11.63, 6.95), 0.617	−2.49 (−9.00, 4.03), 0.453		
Week 40	7.59 (3.92, 11.26)	7.57 (4.19, 10.96)	0.02 (−5.19, 5.23), 0.994	−1.75 (−9.66, 6.17), 0.656	0.79 (−5.44, 7.02), 0.802		
Overall effect	5.55 (0.38, 10.73)	6.74 (3.06, 10.42)	−1.18 (−6.23, 3.86), 0.642	−2.04 (−9.51, 5.42), 0.583	−0.85 (−6.75, 5.05), 0.777		

*SF-36 (BP)*						0.65	0.94
Week 16	2.65 (−0.75, 6.06)	0.24 (−3.34, 3.82)	2.41 (−2.51, 7.34), 0.337	3.24 (−9.63, 16.10), 0.613	1.13 (−4.55, 6.81), 0.696		
Week 40	2.61 (−1.30, 6.52)	1.15 (−3.13, 5.42)	1.46 (−4.49, 7.42), 0.628	1.45 (−12.87, 15.76), 0.840	0.22 (−6.36, 6.80), 0.948		
Overall effect	2.63 (−0.83, 6.09)	0.69 (−3.07, 4.46)	1.94 (−2.60, 6.48), 0.402	2.34 (−9.58, 14.26), 0.691	0.68 (−4.63, 5.98), 0.803		

*SF-36 (GH)*						0.99	0.84
Week 16	3.60 (0.58, 6.62)	6.18 (3.26, 9.11)	−2.59 (−6.70, 1.52), 0.218	−1.64 (−10.49, 7.21), 0.716	−3.01 (−7.89, 1.87), 0.227		
Week 40	8.81 (5.74, 11.88)	6.74 (3.66, 9.82)	2.07 (−2.17, 6.31), 0.339	2.17 (−6.65, 10.99), 0.628	2.21 (−3.06, 7.48), 0.410		
Overall effect	6.20 (0.63, 11.78)	6.46 (3.53, 9.40)	−0.26 (−4.04, 3.52), 0.893	0.27 (−7.76, 8.30), 0.948	−0.40 (−5.02, 4.23), 0.866		

*SF-36 (VT)*						0.55	0.69
Week 16	2.92 (0.93, 4.91)	3.33 (1.25, 5.42)	−0.41 (−3.31, 2.49), 0.780	−2.37 (−7.93, 3.19), 0.397	−0.09 (−3.74, 3.56), 0.962		
Week 40	7.34 (5.14, 9.53)	4.98 (2.72, 7.24)	2.36 (−0.78, 5.49), 0.141	1.20 (−5.84, 8.24), 0.738	2.15 (−1.58, 5.89), 0.258		
Overall effect	5.13 (0.61, 9.65)	4.16 (1.63, 6.69)	0.97 (−1.69, 3.63), 0.473	−0.58 (−6.16, 4.99), 0.836	1.03 (−2.28, 4.35), 0.540		

*SF-36 (SF)*						0.29	0.33
Week 16	4.23 (1.28, 7.18)	3.36 (0.29, 6.44)	0.87 (−3.18, 4.92), 0.673	3.70 (−4.60, 11.99), 0.382	−0.37 (−5.24, 4.51), 0.883		
Week 40	4.42 (1.15, 7.69)	3.13 (−0.05, 6.31)	1.29 (−3.32, 5.90), 0.583	0.57 (−9.42, 10.57), 0.910	1.87 (−3.28, 7.02), 0.477		
Overall effect	4.33 (1.36, 7.29)	3.25 (0.28, 6.22)	1.08 (−2.68, 4.84), 0.573	2.14 (−5.45, 9.72), 0.580	0.75 (−3.76, 5.26), 0.744		

*SF-36 (RE)*						0.32	0.18
Week 16	8.63 (3.78, 13.47)	8.78 (3.94, 13.63)	−0.16 (−7.15, 6.84), 0.964	−4.03 (−0.60, 12.53), 0.633	1.92 (−5.77, 9.60), 0.625		
Week 40	8.93 (4.05, 13.81)	10.70 (5.60, 15.80)	−1.76 (−8.91, 5.38), 0.628	3.63 (−13.52, 20.77), 0.678	−2.98 (−12.27, 6.31), 0.523		
Overall effect	8.78 (4.15, 13.41)	9.74 (4.73, 14.75)	−0.96 (−7.08, 5.15), 0.758	−0.20 (−3.98, 13.57), 0.977	−0.53 (−8.09, 7.03), 0.889		

*SF-36 (MH)*						0.31	0.94
Week 16	2.97 (0.53, 5.41)	4.12 (1.73, 6.51)	−1.15 (−4.66, 2.36), 0.518	−3.90 (−10.24, 2.44), 0.226	0.23 (−3.87, 4.33), 0.912		
Week 40	6.69 (3.83, 9.54)	5.31 (2.54, 8.08)	1.37 (−2.51, 5.25), 0.487	−1.78 (−9.28, 5.72), 0.641	2.94 (−1.59, 7.48), 0.203		
Overall effect	4.83 (0.62, 9.03)	4.72 (2.03, 7.40)	0.11 (−3.26, 3.48), 0.948	−2.84 (−9.08, 3.40), 0.370	1.59 (−2.33, 5.50), 0.427		
HAD	—	—	—	—	—	—	—

*Anxiety*						0.73	0.98
Week 16	−0.68 (−1.17, −0.18)	−0.67 (−1.16, −0.18)	−0.01 (−0.72, 0.70), 0.979	0.02 (−1.28, 1.32), 0.974	−0.20 (−1.05, 0.65), 0.642		
Week 40	−1.49 (−1.98, −1.00)	−1.43 (−1.93, −0.93)	−0.06 (−0.77, 0.64), 0.864	0.01 (−1.37, 1.40), 0.987	−0.26 (−1.14, 0.62), 0.553		
Overall effect	−1.08 (−1.96, −0.21)	−1.05 (−1.89, −0.21)	−0.04 (−0.66, 0.59), 0.911	0.02 (−1.14, 1.18), 0.977	−0.23 (−0.99, 0.53), 0.546		

*Depression*						0.61	0.71
Week 16	−0.92 (−1.38, −0.47)	−0.73 (−1.20, −0.27)	−0.19 (−0.84, 0.46), 0.565	0.09 (−1.09, 1.28), 0.879	−0.21 (−0.98, 0.57), 0.600		
Week 40	−1.82 (−2.27, −1.37)	−1.33 (−1.76, −0.89)	−0.49 (−1.14, 0.15), 0.134	−0.52 (−1.55, 0.52), 0.330	−0.41 (−1.12, 0.29), 0.251		
Overall effect	−1.37 (−2.29, −0.45)	−1.03 (−1.71, −0.34)	−0.34 (−0.88, 0.20), 0.217	−0.21 (−1.08, 0.66), 0.631	−0.31 (−0.97, 0.35), 0.355		

*SES*						0.61	0.79
Week 16	0.97 (0.14, 1.79)	0.77 (−0.03, 1.57)	0.20 (−0.93, 1.32), 0.733	−0.18 (−2.34, 1.99), 0.869	0.34 (−1.04, 1.72), 0.626		
Week 40	2.71 (1.89, 3.53)	1.65 (0.70, 2.61)	1.05 (−0.18, 2.29), 0.095	0.26 (−2.45, 2.96), 0.844	1.42 (−0.07, 2.91), 0.062		
Overall effect	1.84 (0.06, 3.62)	1.21 (0.07, 2.35)	0.63 (−0.41, 1.66), 0.236	0.04 (−2.19, 2.27), 0.971	0.88 (−0.41, 2.17), 0.179		

^1^Percentage of reduction from baseline for weight, BMI, hipline, WHR, and PBF: (before value − after value)/before value ^∗^ 100%; change from baseline for IWQOL-Lite, SF-36, and HAD score: after value (posttreatment) – before value (baseline). ^2^*P* value for interaction between sex and treatment group. ^3^*P* value for interaction among sex, treatment group, and visit time point. ^*∗*^The average change during the total follow-up period was estimated as the overall effect. BMI, body mass index; WHR, waist-to-hip ratio; PBF, percent body fat; IWQOL-Lite, Impact of Weight on Quality of Life-Lite; HAD, Hospital Anxiety and Depression Scale; SF-36, 36-Item Short Form; SES, Self-Esteem Scale.

**Table 8:** Secondary outcomes (change from baseline) based on the intention-to-treat principle with multiple imputation.

	Adjusted mean^1^			Difference (95% CI), *P*		*P* ^2^	*P* ^3^
	ACE group (95% CI)	Sham ACE group (95% CI)	Difference (95% CI), *P*	Male group (*n* = 51)	Female group (*n* = 165)		
*Weight (*kg)						0.62	0.46
Baseline	74.93 ± 9.44	74.00 ± 10.01	—	—	—		
Change at week 16	−4.17 (−4.71, −3.63)	−1.85 (−2.47, −1.22)	−2.32 (−3.15, −1.49), <0.001	−2.26 (−4.69, 0.17), 0.068	−3.85 (−4.88, −2.82), <0.001		
Change at week 40	−5.38 (−6.08, −4.69)	−1.86 (−2.68, −1.03)	−3.53 (−4.67, −2.39), <0.001	−2.07 (−4.82, 0.68), 0.139	−3.67 (−4.82, −2.51), <0.001		
Overall change	−4.19 (−7.37, −1.00)	−1.70 (−2.67, −0.74)	−2.48 (−3.22, −1.75), <0.001	−1.51 (−3.41, 0.39), 0.118	−2.60 (−3.38, −1.82), <0.001		

*BMI (*kg/m^2^)						0.62	0.49
Baseline	27.40 ± 1.62	27.49 ± 1.79	—	—	—		
Change at week 16	−1.55 (−1.75, −1.35)	−0.73 (−0.93, −0.54)	−0.82 (−1.10, −0.54), <0.001	−0.84 (−1.51, −0.18), 0.013	−1.49 (−1.88, −1.11), <0.001		
Change at week 40	−2.00 (−2.25, −1.74)	−0.76 (−1.01, −0.50)	−1.24 (−1.61, −0.88), <0.001	−0.78 (−1.48, −0.07), 0.032	−1.40 (−1.80, −1.00), <0.001		
Overall change	−1.55 (−2.73, −0.37)	−0.68 (−1.04, −0.32)	−0.88 (−1.12, −0.63), <0.001	−0.57 (−1.06, −0.08), 0.022	−1.00 (−1.29, −0.71), <0.001		

*Hipline (cm)*						0.89	0.98
Baseline	104.16 ± 6.07	103.74 ± 6.67	—	—	—		
Change at week 16	−2.72 (−3.25, −2.20)	−1.59 (−2.14, −1.03)	−1.14 (−1.91, −0.37), 0.004	−1.14 (−3.12, 0.85), 0.260	−2.24 (−3.27, −1.21), <0.001		
Change at week 40	−3.94 (−4.76, −3.13)	−1.80 (−2.63, −0.98)	−2.14 (−3.28, −1.00), <0.001	−1.69 (−5.02, 1.64), 0.320	−2.25 (−3.47, −1.04), <0.001		
Overall change	−2.88 (−5.20, −0.56)	−1.58 (−2.73, −0.42)	−1.30 (−2.00, −0.60), <0.001	−0.70 (−2.31, 0.91), 0.393	−1.56 (−2.34, −0.79), <0.001		

*WHR*						0.36	0.44
Baseline	89.61 ± 5.71	89.55 ± 5.78	—	—	—		
Change at week 16	−3.01 (−3.69, −2.32)	−1.92 (−2.64, −1.21)	−1.08 (−2.10, −0.07), 0.036	−2.68 (−4.73, −0.64), 0.010	−2.45 (−3.67, −1.23), <0.001		
Change at week 40	−4.24 (−5.03, −3.45)	−1.43 (−2.23, −0.63)	−2.81 (−3.94, −1.69), <0.001	−2.10 (−4.62, 0.42), 0.102	−2.54 (−3.90, −1.18), <0.001		
Overall change	−3.17 (−5.75, −0.60)	−1.58 (−2.47, −0.68)	−1.59 (−2.42, −0.77), <0.001	−1.35 (−3.08, 0.38), 0.126	−1.31 (−2.28, −0.34), 0.008		

*PBF*						*0.78*	*0.54*
Baseline	32.32 ± 4.03	32.69 ± 3.67	—	—	—		
Change at week 16	−2.32 (−2.70, −1.93)	−1.69 (−2.08, −1.29)	−0.63 (−1.17, −0.09), 0.023	−0.98 (−2.84, 0.88), 0.297	−1.84 (−2.53, −1.16), <0.001		
Change at week 40	−2.71 (−3.21, −2.21)	−1.32 (−1.83, −0.81)	−1.39 (−2.12, −0.66), <0.001	−0.86 (−2.72, 1.01), 0.364	−1.68 (−2.45, −0.90), <0.001		
Overall change	−2.28 (−3.83, −0.73)	−1.36 (−2.10, −0.61)	−0.92 (−1.41, −0.42), <0.001	−0.72 (−2.15, 0.70), 0.318	−1.03 (−1.54, −0.52), <0.001		

*IWQOL-Lite*						0.96	0.25
Baseline	58.43 ± 16.92	56.71 ± 17.34	—	—	—		
Change at week 16	−11.19 (−13.68, −8.71)	−8.96 (−11.66, −6.25)	−2.24 (−6.05, 1.58), 0.248	2.43 (−3.96, 8.81), 0.456	−3.46 (−7.67, 0.74), 0.106		
Change at week 40	−16.72 (−19.15, −14.29	−10.37 (−12.75, −7.98)	−6.35 (−9.72, −2.99), <0.001	0.20 (−5.02, 5.42), 0.940	−8.42 (−12.37, −4.47), <0.001		
Overall change	−10.73 (−19.44, −2.03)	−8.13 (−13.22, −3.05)	−2.60 (−5.14, −0.06), 0.045	1.48 (−2.30, 5.26), 0.443	−3.81 (−6.80, −0.82), 0.013		
SF-36 score	—	—	—	—	—		

*SF-36 (PF)*						0.20	0.65
Baseline	89.35 ± 10.26	90.93 ± 9.67	—	—	—		
Change at week 16	2.90 (1.12, 4.68)	0.93 (−0.85, 2.71)	1.97 (−0.54, 4.48), 0.124	−1.85 (−5.53, 1.83), 0.323	3.04 (−0.00, 6.09), 0.050		
Change at week 40	4.45 (2.89, 6.00)	2.92 (1.36, 4.49)	1.53 (−0.70, 3.75), 0.180	−0.85 (−6.09, 4.38), 0.748	2.40 (−0.14, 4.94), 0.064		
Overall change	3.67 (1.56, 5.79)	1.93 (−0.48, 4.34)	1.75 (−0.43, 3.92), 0.115	−1.35 (−5.27, 2.57), 0.497	2.72 (0.15, 5.29), 0.038		

*SF-36 (RP)*						0.59	0.52
Baseline	84.03 ± 26.79	88.89 ± 22.76	—	—	—		
Change at week 16	3.52 (−0.53, 7.56)	5.91 (2.29, 9.52)	−2.39 (−8.04, 3.26), 0.404	−2.34 (−11.63, 6.95), 0.617	−2.49 (−9.00, 4.03), 0.453		
Change at week 40	7.59 (3.92, 11.26)	7.57 (4.19, 10.96)	0.02 (−5.19, 5.23), 0.994	−1.75 (−9.66, 6.17), 0.656	0.79 (−5.44, 7.02), 0.802		
Overall change	5.55 (0.38, 10.73)	6.74 (3.06, 10.42)	−1.18 (−6.23, 3.86), 0.642	−2.04 (−9.51, 5.42), 0.583	−0.85 (−6.75, 5.05), 0.777		

*SF-36 (BP)*						0.65	0.94
Baseline	77.98 ± 19.92	78.81 ± 21.38	—	—	—		
Change at week 16	2.65 (−0.75, 6.06)	0.24 (−3.34, 3.82)	2.41 (−2.51, 7.34), 0.337	3.24 (−9.63, 16.10), 0.613	1.13 (−4.55, 6.81), 0.696		
Change at week 40	2.61 (−1.30, 6.52)	1.15 (−3.13, 5.42)	1.46 (−4.49, 7.42), 0.628	1.45 (−12.87, 15.76), 0.840	0.22 (−6.36, 6.80), 0.948		
Overall change	2.63 (−0.83, 6.09)	0.69 (−3.07, 4.46)	1.94 (−2.60, 6.48), 0.402	2.34 (−9.58, 14.26), 0.691	0.68 (−4.63, 5.98), 0.803		

*SF-36 (GH)*						0.99	0.84
Baseline	61.69 ± 19.03	66.40 ± 15.16	—	—	—		
Change at week 16	3.60 (0.58, 6.62)	6.18 (3.26, 9.11)	−2.59 (−6.70, 1.52), 0.218	−1.64 (−10.49, 7.21), 0.716	−3.01 (−7.89, 1.87), 0.227		
Change at week 40	8.81 (5.74, 11.88)	6.74 (3.66, 9.82)	2.07 (−2.17, 6.31), 0.339	2.17 (−6.65, 10.99), 0.628	2.21 (−3.06, 7.48), 0.410		
Overall change	6.20 (0.63, 11.78)	6.46 (3.53, 9.40)	−0.26 (−4.04, 3.52), 0.893	0.27 (−7.76, 8.30), 0.948	−0.40 (−5.02, 4.23), 0.866		

*SF-36 (VT)*						0.55	0.69
Baseline	73.19 ± 13.83	73.33 ± 13.53	—	—	—		
Change at week 16	2.92 (0.93, 4.91)	3.33 (1.25, 5.42)	−0.41 (−3.31, 2.49), 0.780	−2.37 (−7.93, 3.19), 0.397	−0.09 (−3.74, 3.56), 0.962		
Change at week 40	7.34 (5.14, 9.53)	4.98 (2.72, 7.24)	2.36 (−0.78, 5.49), 0.141	1.20 (−5.84, 8.24), 0.738	2.15 (−1.58, 5.89), 0.258		
Overall change	5.13 (0.61, 9.65)	4.16 (1.63, 6.69)	0.97 (−1.69, 3.63), 0.473	−0.58 (−6.16, 4.99), 0.836	1.03 (−2.28, 4.35), 0.540		

*SF-36 (SF)*						0.29	0.33
Baseline	93.87 ± 19.82	94.68 ± 19.05	—	—	—		
Change at week 16	4.23 (1.28, 7.18)	3.36 (0.29, 6.44)	0.87 (−3.18, 4.92), 0.673	3.70 (−4.60, 11.99), 0.382	−0.37 (−5.24, 4.51), 0.883		
Change at week 40	4.42 (1.15, 7.69)	3.13 (−0.05, 6.31)	1.29 (−3.32, 5.90), 0.583	0.57 (−9.42, 10.57), 0.910	1.87 (−3.28, 7.02), 0.477		
Overall change	4.33 (1.36, 7.29)	3.25 (0.28, 6.22)	1.08 (−2.68, 4.84), 0.573	2.14 (−5.45, 9.72), 0.580	0.75 (−3.76, 5.26), 0.744		

*SF-36 (RE)*						0.32	0.18
Baseline	78.40 ± 31.68	76.23 ± 31.59	—	—	—		
Change at week 16	8.63 (3.78, 13.47)	8.78 (3.94, 13.63)	−0.16 (−7.15, 6.84), 0.964	−4.03 (−0.60, 12.53), 0.633	1.92 (−5.77, 9.60), 0.625		
Change at week 40	8.93 (4.05, 13.81)	10.70 (5.60, 15.80)	−1.76 (−8.91, 5.38), 0.628	3.63 (−13.52, 20.77), 0.678	−2.98 (−12.27, 6.31), 0.523		
Overall change	8.78 (4.15, 13.41)	9.74 (4.73, 14.75)	−0.96 (−7.08, 5.15), 0.758	−0.20 (−3.98, 13.57), 0.977	−0.53 (−8.09, 7.03), 0.889		

*SF-36 (MH)*						0.31	0.94
Baseline	71.63 ± 12.99	70.67 ± 13.01	—	—	—		
Change at week 16	2.97 (0.53, 5.41)	4.12 (1.73, 6.51)	−1.15 (−4.66, 2.36), 0.518	−3.90 (−10.24, 2.44), 0.226	0.23 (−3.87, 4.33), 0.912		
Change at week 40	6.69 (3.83, 9.54)	5.31 (2.54, 8.08)	1.37 (−2.51, 5.25), 0.487	−1.78 (−9.28, 5.72), 0.641	2.94 (−1.59, 7.48), 0.203		
Overall change	4.83 (0.62, 9.03)	4.72 (2.03, 7.40)	0.11 (−3.26, 3.48), 0.948	−2.84 (−9.08, 3.40), 0.370	1.59 (−2.33, 5.50), 0.427		
HAD score							

*Anxiety*						0.73	0.98
Baseline	4.21 ± 2.82	4.58 ± 2.90	—	—	—		
Change at week 16	−0.68 (−1.17, −0.18)	−0.67 (−1.16, −0.18)	−0.01 (−0.72, 0.70), 0.979	0.02 (−1.28, 1.32), 0.974	−0.20 (−1.05, 0.65), 0.642		
Change at week 40	−1.49 (−1.98, −1.00)	−1.43 (−1.93, −0.93)	−0.06 (−0.77, 0.64), 0.864	0.01 (−1.37, 1.40), 0.987	−0.26 (−1.14, 0.62), 0.553		
Overall change	−1.08 (−1.96, −0.21)	−1.05 (−1.89, −0.21)	−0.04 (−0.66, 0.59), 0.911	0.02 (−1.14, 1.18), 0.977	−0.23 (−0.99, 0.53), 0.546		

*Depression*						0.61	0.71
Baseline	4.29 ± 2.74	3.87 ± 2.88	—	—	—		
Change at week 16	−0.92 (−1.38, −0.47)	−0.73 (−1.20, −0.27)	−0.19 (−0.84, 0.46), 0.565	0.09 (−1.09, 1.28), 0.879	−0.21 (−0.98, 0.57), 0.600		
Change at week 40	−1.82 (−2.27, −1.37)	−1.33 (−1.76, −0.89)	−0.49 (−1.14, 0.15), 0.134	−0.52 (−1.55, 0.52), 0.330	−0.41 (−1.12, 0.29), 0.251		
Overall change	−1.37 (−2.29, −0.45)	−1.03 (−1.71, −0.34)	−0.34 (−0.88, 0.20), 0.217	−0.21 (−1.08, 0.66), 0.631	−0.31 (−0.97, 0.35), 0.355		

*SES*						0.61	0.79
Baseline	32.41 ± 4.01	32.65 ± 4.75	—	—	—		
Change at week 16	0.97 (0.14, 1.79)	0.77 (−0.03, 1.57)	0.20 (−0.93, 1.32), 0.733	−0.18 (−2.34, 1.99), 0.869	0.34 (−1.04, 1.72), 0.626		
Change at week 40	2.71 (1.89, 3.53)	1.65 (0.70, 2.61)	1.05 (−0.18, 2.29), 0.095	0.26 (−2.45, 2.96), 0.844	1.42 (−0.07, 2.91), 0.062		
Overall change	1.84 (0.06, 3.62)	1.21 (0.07, 2.35)	0.63 (−0.41, 1.66), 0.236	0.04 (−2.19, 2.27), 0.971	0.88 (−0.41, 2.17), 0.179		

^1^Change from baseline: after value (posttreatment) – before value (baseline); ^2^*P* value for interaction between sex and treatment group; ^3^*P* value for interaction among sex, treatment group, and visit time point. BMI, body mass index; WHR, waist-to-hip ratio; PBF, percent body fat; IWQOL-Lite, Impact of Weight on Quality of Life-Lite; HAD, Hospital Anxiety and Depression Scale; SF-36, 36-Item Short Form; SES, Self-Esteem Scale.

**Table 9:** Outcomes at baseline and the end of study.

Outcomes	ACE group (*n* = 108)	Sham ACE group (*n* = 108)	*Z*	*P*
*Waistline, median (IQR) (cm)*				
Baseline	93.25 (87.50, 99.00)	92.00 (88.00, 98.00)	−0.314	0.754
End of treatment (16th week)	85.00 (79.00, 90.00)	89.00 (84.00, 95.00)	4.135	<0.001
End of study (40th week)	85.00 (80.00, 90.00)	89.00 (85.00, 95.00)	4.531	<0.001

*Weight, median (IQR) (kg)*				
Baseline	72.90 (67.45, 80.25)	72.00 (66.30, 80.60)	−0.710	0.478
End of treatment (16th week)	66.15 (61.30, 76.00)	69.60 (65.00, 79.10)	2.490	0.013
End of study (40th week)	67.50 (61.50, 76.00)	70.80 (65.20, 80.00)	2.125	0.034

*BMI, median (IQR) (kg/m* ^*2*^)				
Baseline	27.42 (25.97, 28.89)	27.49 (25.95, 28.93)	0.186	0.852
End of treatment (16th week)	25.11 (23.85, 26.50)	26.62 (25.59, 28.16)	5.039	<0.001
End of study (40th week)	25.39 (23.74, 26.92)	26.83 (25.37, 28.16)	4.935	<0.001

*Hipline, median (IQR) (cm)*				
Baseline	104.00 (100.00, 108.00)	103.00 (100.00, 107.50)	−0.698	0.485
End of treatment (16th week)	99.45 (96.50, 104.00)	100.00 (98.00, 105.00)	1.894	0.058
End of study (40th week)	100.00 (96.00, 103.00)	101.00 (97.30, 105.50)	2.210	0.027

*WHR, median (IQR)*				
Baseline	90.00 (85.00, 95.00)	90.00 (86.00, 94.00)	0.151	0.880
End of treatment (16th week)	85.00 (81.00, 89.00)	88.00 (84.00, 92.00)	3.298	<0.001
End of study (40th week)	85.50 (80.00, 90.00)	88.00 (85.00, 92.00)	3.250	0.001

*PBF, median (IQR) (%)*				
Baseline	33.10 (30.25, 35.10)	33.15 (30.50, 35.35)	0.420	0.674
End of treatment (16th week)	29.35 (26.50, 32.00)	31.70 (29.50, 34.00)	3.930	<0.001
End of study (40th week)	29.90 (27.10, 32.00)	32.00 (28.50, 34.60)	3.702	<0.001

*IWQOL-Lite, median (IQR)*				
Baseline	58.00 (44.50, 72.00)	53.50 (44.00, 66.50)	−1.045	0.296
End of treatment (16th week)	39.00 (35.00, 60.00)	44.00 (35.00, 59.00)	−0.115	0.908
End of study (40th week)	37.00 (33.00, 48.00)	42.00 (33.00, 58.00)	−1.855	0.064
SF-36, median (IQR)				

*PF*				
Baseline	95.00 (85.00, 95.00)	95.00 (90.00, 95.00)	1.206	0.228
End of treatment (16th week)	95.00 (90.00, 100.00)	95.00 (90.00, 100.00)	0.648	0.517
End of study (40th week)	95.00 (90.00, 100.00)	95.00 (90.00, 100.00)	1.289	0.197

*RP*				
Baseline	100.00 (75.00, 100.00)	100.00 (75.00, 100.00)	1.421	0.155
End of treatment (16th week)	100.00 (100.00, 100.00)	100.00 (100.00, 100.00)	−0.217	0.828
End of study (40th week)	100.00 (100.00, 100.00)	100.00 (100.00, 100.00)	0.675	0.500

*BP*				
Baseline	72.00 (62.00, 100.00)	72.00 (62.00, 100.00)	0.145	0.885
End of treatment (16th week)	80.00 (72.00, 100.00)	72.00 (62.00, 100.00)	1.832	0.067
End of study (40th week)	100.00 (62.00, 100.00)	100.00 (62.00, 100.00)	0.869	0.385

*GH*				
Baseline	62.00 (48.50, 76.00)	67.00 (57.00, 77.00)	1.679	0.093
End of treatment (16th week)	67.00 (52.00, 77.00)	72.00 (58.50, 82.00)	−1.067	0.286
End of study (40th week)	72.00 (62.00, 82.00)	67.00 (60.00, 85.00)	0.643	0.520

*VT*				
Baseline	75.00 (65.00, 80.00)	75.00 (65.00, 80.00)	0.144	0.886
End of treatment (16th week)	80.00 (70.00, 85.00)	80.00 (70.00, 85.00)	−0.535	0.593
End of study (40th week)	80.00 (70.00, 85.00)	80.00 (70.00, 85.00)	0.623	0.534

*SF*				
Baseline	100.00 (87.50, 112.50)	100.00 (87.50, 112.50)	0.196	0.844
End of treatment (16th week)	100.00 (87.50, 112.50)	100.00 (87.50, 112.50)	0.126	0.900
End of study (40th week)	100.00 (87.50, 112.50)	100.00 (87.50, 112.50)	0.017	0.986

*RE*				
Baseline	100.00 (66.67, 100.00)	100.00 (66.67, 100.00)	−0.724	0.469
End of treatment (16th week)	100.00 (100.00, 100.00)	100.00 (83.33, 100.00)	0.701	0.483
End of study (40th week)	100.00 (100.00, 100.00)	100.00 (100.00, 100.00)	−0.015	0.988

*MH*				
Baseline	72.00 (64.00, 80.00)	72.00 (64.00, 80.00)	−0.545	0.586
End of treatment (16th week)	72.00 (68.00, 80.00)	76.00 (66.00, 84.00)	−0.604	0.546
End of study (40th week)	76.00 (68.00, 88.00)	76.00 (68.00, 88.00)	0.741	0.459
HAD, median (IQR)				

*Anxiety*				
Baseline	4.00 (2.00, 6.00)	4.00 (3.00, 6.00)	1.039	0.299
End of treatment (16th week)	3.00 (2.00, 5.00)	3.00 (2.00, 6.00)	−0.801	0.423
End of study (40th week)	2.00 (1.00, 4.00)	3.00 (1.00, 5.00)	−1.083	0.279

*Depression*				
Baseline	4.00 (2.00, 6.00)	3.00 (2.00, 5.50)	−1.240	0.215
End of treatment (16th week)	3.00 (1.00, 4.00)	3.00 (1.50, 5.00)	−0.574	0.566
End of study (40th week)	2.00 (1.00, 3.00)	2.00 (1.00, 4.00)	−1.610	0.107

*SES, median (IQR)*				
Baseline	33.00 (30.00, 35.00)	32.50 (29.00, 36.50)	0.294	0.769
End of treatment (16th week)	33.00 (30.00, 36.00)	33.00 (30.00, 38.00)	0.416	0.677
End of study (40th week)	35.00 (32.00, 39.00)	34.00 (30.00, 39.00)	1.663	0.096

BMI, body mass index; WHR, waist-to-hip ratio; PBF, percent body fat; IWQOL-Lite, Impact of Weight on Quality of Life-Lite; HAD, Hospital Anxiety and Depression Scale; SF-36, 36-Item Short Form; SES, Self-Esteem Scale.

**Table 10:** Blinding assessment results.

	At week 8		At week 16	
Patients' answers	ACE(*n* = 101)	Sham ACE(*n* = 99)	ACE(*n* = 98)	Sham ACE(*n* = 97)
“ACE,” *n* (%)	93 (92.1)	89 (89.9)	92 (93.9)	89 (91.8)
“Sham ACE,” *n* (%)	8 (7.9)	10 (10.1)	6 (6.1)	8 (8.2)
*P* value^*∗*^	<0.001	<0.001		

ACE = acupoint catgut embedding; Sham ACE = sham acupoint catgut embedding; ^*∗*^McNemar test.

**Table 11:** Adverse events related to treatment^a^.

Adverse event	Participant, No. (%)	
	ACE group (*n* = 106)^b^	Sham ACE group(*n* = 107)^b^
Overall	8 (7.5%)	5 (4.7%)
Severe adverse events	0	0
Sleeplessness after ACE	1 (0.9%)	0
Dizziness after ACE	1 (0.9%)	1 (0.9%)
Fainting during ACE	1 (0.9%)	0 (0)
Nausea during ACE	0 (0)	0 (0)
Local induration after two weeks	0 (0)	0 (0)
Haematoma around the site of needling	2 (1.9%)	0 (0)
Sharp pain lasting＞2h^c^	1 (0.9%)	3 (2.8%)
Bleeding/numbness/infection around the site of needling	0 (0)	0 (0)
Other discomforts after ACE^d^	2 (1.9%)	1 (0.9%)

^a^Adverse events were analysed in all participants who received treatment. Adverse events were counted by type rather than the frequency in the same participant. Adverse events with different types occurring in a single participant were defined as independent adverse events. An adverse event with multiple occurrences in a single participant was defined as 1 adverse event. ^b^Two participants in the ACE group and one in the sham ACE group did not receive treatment. ^c^Sharp pain was defined as VAS ≥4.^d^ Include headache, local edema, transient weakness of the hand, and mild pain.

**Table 12:** Safety outcomes based on per-protocol analysis: laboratory tests.

Indictors	Time point	Abnormal (%)		*P*
		ACE group	Sham ACE group	
WBC	Baseline	7 (6.54%)	2 (1.85%)	0.169
	16 weeks	4 (4.17%)	1 (1.01%)	0.347
NEU	Baseline	2 (1.87%)	3 (2.78%)	1.00
	16 weeks	1 (1.04%)	2 (2.02%)	1.00
LYM	Baseline	5 (4.67%)	6 (5.56%)	0.769
	16 weeks	2 (2.08%)	5 (5.05%)	0.466
RBC	Baseline	20 (18.69%)	17 (15.74%)	0.567
	16 weeks	17 (17.71%)	16 (16.16%)	0.773
HGB	Baseline	25 (23.36%)	19 (17.59%)	0.294
	16 weeks	22 (22.92%)	20 (20.20%)	0.645
PLT	Baseline	6 (5.61%)	3 (2.78%)	0.487
	16 weeks	6 (6.25%)	6 (6.06%)	0.956
ALT	Baseline	15 (13.89%)	16 (14.81%)	0.846
	16 weeks	14 (14.29%)	9 (9.09%)	0.256
AST	Baseline	12 (11.11%)	9 (8.33%)	0.491
	16 weeks	11 (11.22%)	9 (9.09%)	0.620
GGT	Baseline	7 (8.33%)	6 (7.14%)	0.773
	16 weeks	5 (5.38%)	9 (9.28%)	0.303
ALP	Baseline	4 (4.76%)	7 (8.33%)	0.349
	16 weeks	5 (5.32%)	7 (7.14%)	0.602
T-BIL	Baseline	16 (14.81%)	16 (14.81%)	1.000
	16 weeks	14 (14.43%)	15 (15.31%)	0.864
BUN	Baseline	4 (3.70%)	6 (5.56%)	0.517
	16 weeks	7 (7.14%)	3 (3.03%)	0.322
Cr	Baseline	28 (25.93%)	14 (12.96%)	0.016
	16 weeks	21 (21.43%)	15 (15.15%)	0.254

WBC, white blood cell; NEU, neutrophil percentage; LYM, lymphocyte percentage; RBC, red blood cell; HGB, hemoglobin; PLT, platelet; ALT, alanine aminotransferase; AST, aspartic acid aminotransferase; GGT, gamma-glutamyl transferase; ALP, alkaline phosphatase; T-BIL, total bilirubin; BUN, urea nitrogen; Cr, creatinine.

## Data Availability

The data collected and/or analysed during this study are available from the corresponding author on reasonable request.

## Abbreviations

ACE: Acupoint catgut embedding
CI: Confidence interval
SOPs: Standard operating procedures
BMI: Body mass index
WHR: Waist-to-hip ratio
PBF: Percent body fat
IWQOL-Lite: Impact of Weight on Quality of Life-Lite
SF-36: 36-Item Short Form
HAD: Hospital Anxiety and Depression Scale
SES: Self-Esteem Scale
AEs: Adverse events
VAS: Visual analogue scale
IQR: Interquartile range
ITT: Intention-to-treat
WBC: White blood cell
NEU: Neutrophil percentage
LYM: Lymphocyte percentage
RBC: Red blood cell
HGB: Hemoglobin
PLT: Platelet
ALT: Alanine aminotransferase
AST: Aspartic acid aminotransferase
GGT: Gamma-glutamyl transferase
ALP: Alkaline phosphatase
T-BIL: Total bilirubin
BUN: Urea nitrogen
Cr: Creatinine
PROC MI: Procedure multiple imputation
FCS: Fully conditional specification.
